# Impact of epidermal growth factor receptor sensitizing mutations on outcomes of patients with non-small cell lung cancer treated with definitive thoracic radiation therapy: a systematic review and meta-analysis

**DOI:** 10.18632/oncotarget.21019

**Published:** 2017-09-18

**Authors:** Yu Yang Soon, Balamurugan Vellayappan, Jeremy Chee Seong Tey, Cheng Nang Leong, Wee Yao Koh, Ivan Weng Keong Tham

**Affiliations:** ^1^ Department of Radiation Oncology, National University Cancer Institute, Singapore; ^2^ National University Hospital, Singapore; ^3^ National University of Singapore, Singapore

**Keywords:** non-small cell lung cancer, epidermal growth factor receptor, radiotherapy, chemo-radiotherapy, prognosis

## Abstract

**Background:**

To determine if the presence of epidermal growth factor receptor (EGFR) sensitizing mutations improves tumor control and survival outcomes in patients with non-metastatic non-small cell lung cancer (NSCLC) who received definitive thoracic radiation therapy (TRT) with or without chemotherapy.

**Materials and Methods:**

We searched MEDLINE for eligible comparative studies which compared the outcomes of patients treated with definitive TRT according to EGFR mutation status. Meta-analysis was performed using random effects model. Outcomes of interest were tumor overall response rate (ORR), loco-regional (LRR), distant recurrence rates (DRR), relapse-free survival (RFS), overall survival (OS) and adverse events (AE).

**Results:**

We found seven studies including 537 patients with stage III NSCLC. Up to 45% of patients in the studies had mutations in exon 19 and 21. Patients harbouring EGFR sensitizing mutations had a trend towards improvement in ORR (risk ratio 1.17, 95% confidence interval 0.99–1.37, *P* = 0.06) compared to EGFR wild type status. There were no significant differences in LRR, DRR, RFS, OS and AE outcomes between the EGFR mutant and EGFR wild type groups.

**Conclusions:**

The presence of EGFR sensitizing mutations may improve tumour response rate but not survival in patients with localized NSCLC treated with definitive thoracic radiation therapy with or without chemotherapy.

## INTRODUCTION

Lung cancer accounted for 1.8 million new cases and 1.59 million deaths worldwide in 2012 [1]. Adenocarcinoma is the most prevalent histologic type [2]. Epidermal growth factor receptor (EGFR) is one of the mutated proto-oncogenes in lung adenocarcinoma, where a sensitizing EGFR mutation can result in constitutive activation of tyrosine kinase (TK) and phosphorylation of downstream pathways leading to uncontrolled proliferation, invasion and metastasis. The frequency of EGFR sensitizing mutations ranges from 15% of lung adenocarcinoma in Caucasian populations to as high as 50% in Asian populations [3–4].

A meta-analysis of 13 randomized trials have shown that EGFR TK inhibitors (TKIs) such as gefitinib or erlotinib delay disease progression significantly but do not improve overall survival compared to first line platinum based chemotherapy in Stage IV lung adenocarcinoma harbouring EGFR sensitizing mutations [5]. The lack of overall survival benefit with EGFR TKIs in these randomized trials is most likely due to the use of these EGFR TKIs as second line therapy after progression on first line chemotherapy [6, 7]. These trials established EGFR TKIs as first line systemic therapy for patients with Stage IV lung adenocarcinoma harbouring EGFR sensitizing mutations [8].

Currently, there is no defined role of EGFR TKIs for patients with Stage I to III lung adenocarcinoma. Definitive thoracic radiation therapy (TRT) with or without chemotherapy remains one of the recommended curative treatment options [8]. Although in-vitro studies suggest that non-small cell lung cancer (NSCLC) cell lines with EGFR mutations have increased sensitivity to radiation compared to EGFR wild-type cell lines [9], it is unclear if patients harbouring these mutations have improved clinical outcomes when treated with definitive TRT compared with patients with EGFR wild-type status. Hence, we performed a systematic review and meta-analysis of comparative studies to determine the impact of EGFR sensitizing mutations on tumour overall response rates, locoregional and distant disease recurrence rates, recurrence-free and overall survival as well as toxicity outcomes in patients with non-metastatic lung adenocarcinoma treated with definitive TRT with or without chemotherapy. The knowledge gained from our findings may help with patient prognostication.

## RESULTS

### Results of search strategy

**Figure 1:**
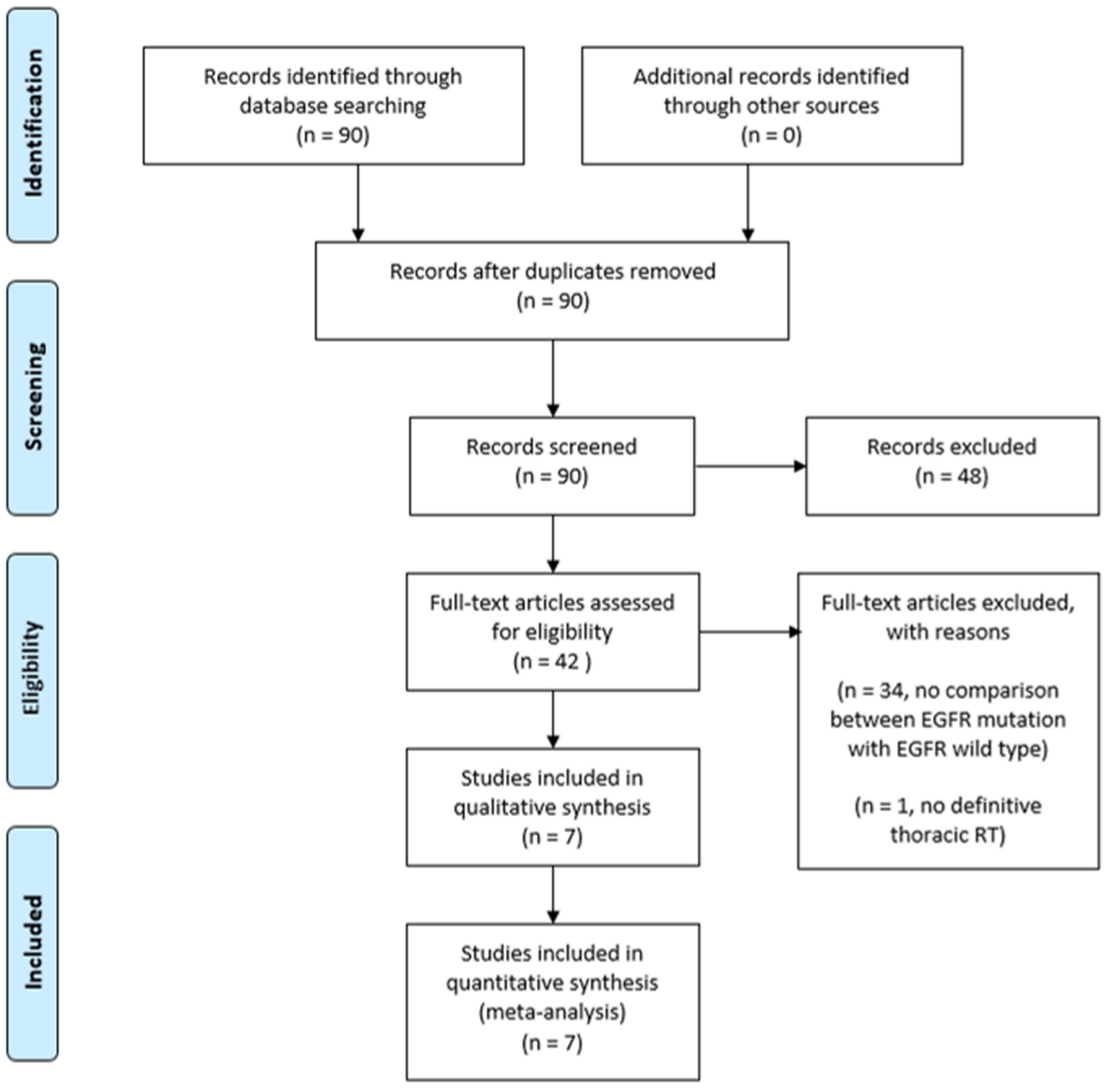
Results of search strategy

We identified seven comparative studies including 537 patients using the search strategy summarized in Figure 1 [10–16]. We screened through 90 records and retrieved 42 full text articles for further assessment. Thirty four articles were excluded as they did not compare the outcomes of the study participants according to their EGFR mutation status. One study was excluded as the participants received adjuvant TRT.

### Characteristics of included studies

The characteristics of the seven included studies are summarized in Table 1. Five of the included studies were single institutional retrospective cohort studies [11–13, 15–16] while the other two were retrospective analysis of prospective single arm clinical trials [10, 14]. The median sample size was 44 (range 34 to 184). Six studies included only patients with stage III disease treated with TRT [10–11, 13–16]. All studies tested for the presence of exon 19 deletion or L858R missense mutation in exon 21. For each study, 10 to 45% of patients had EGFR sensitizing mutations. The reported total thoracic radiation dose among the included studies ranged from 40 to 74 Gy. Only two studies required all patients to receive concurrent platinum doublet chemotherapy [14–15]. There were only two studies that mandated all patients harbouring EGFR sensitizing mutations to receive EGFR TKIs [10, 14].

**Table 1:** Characteristics of included studies

Study	Year published	Study design	Sample size	Median age	Stage III (%)	EGFR mutations tested	EGFR activating mutation (%)	Total Thoracic RT dose received (EQD2) (Gy)	Received concurrent chemo (%)	Systemic therapy regimens	EGFR mutant receiving EGFR TKI (%)	Global score
Ready	2010	Retrospective analysis of prospective single arm trial	43	NR	100	Exon 19 deletions and mutations in exon 18, 20 and 21	26	66	Not reported	Induction phase: 2 cycles of Paclitaxel 200mg/m2 plus Carboplatin area under curve 6 every 21 days plus Gefitinib 250mg daily Concurrent phase: Gefitinib 250mg daily or weekly Paclitaxel 50mg/m2 with Carboplatin area under curve 2 for 7 weeks Maintenance phase: Gefitinib 250mg daily till disease progression or unacceptable side effects	100	B1
Li	2011	Retrospective cohort study	87	NR	100	Exon 19 deletions and mutations in exon 21	45	40-70	59	Not reported	41	B2
Hayashi	2012	Retrospective cohort study	34	69	97	Exon 19 deletions and mutations in exon 18 and 21	32	Not reported	88	Not reported	Not reported	C
Akamatsu	2014	Retrospective cohort study	44	66	100	Exon 19 deletions and mutations in exon 18, 20 and 21	30	56-74	91	Cisplatin plus S-1 or Cisplatin plus Vinorelbine or Carboplatin plus Paclitaxel	77	B2
Komaki	2015	Retrospective analysis of prospective single arm trial	41	NR	100	Exon 19 deletions and mutations in exon 18, 20 and 21	10	60	100	Concurrent phase: weekly Paclitaxel 45mg/m2 plus Carboplatin area under curve 2 for 7 weeks plus Erlotinib 150mg daily from Tuesday to Sunday. Consolidation phase: 2 cycles of Paclitaxel 200mg /m2 plus Carboplatin area under the curve 6 every 21 days	100	B1
Tanaka	2015	Retrospective cohort study	104	62	100	Exon 19 deletions and mutations in exon 18, 20 and 21	28	54-74	100	Carboplatin plus Paclitaxel or Cisplatin plus Vinorelbine or Cisplatin plus Docetaxel or Cisplatin plus S-1 or Cisplatin plus Pemetrexed or Cisplatin plus Irinotecan or Carboplatin plus Vinorelbine	72	B1
Yagaishita	2015	Retrospective cohort study	184	61	100	Exon 19 deletions and mutations in exon 21	16	60	90	Cisplatin plus Vinorelbine or Carboplatin plus Paclitaxel	69	B1

NR: not reported.

Global score: B1, low-moderate risk of bias; B2, moderate-high risk of bias; C, high risk of bias.

Formal critical appraisal of the seven studies showed that the risk of bias was low to moderate in four studies (quality score B1) [10, 14–16], moderate to high in two studies (quality score B2) [10, 13] and high in one study (quality score C) [12].

### Tumour overall response rates

**Figure 2:**
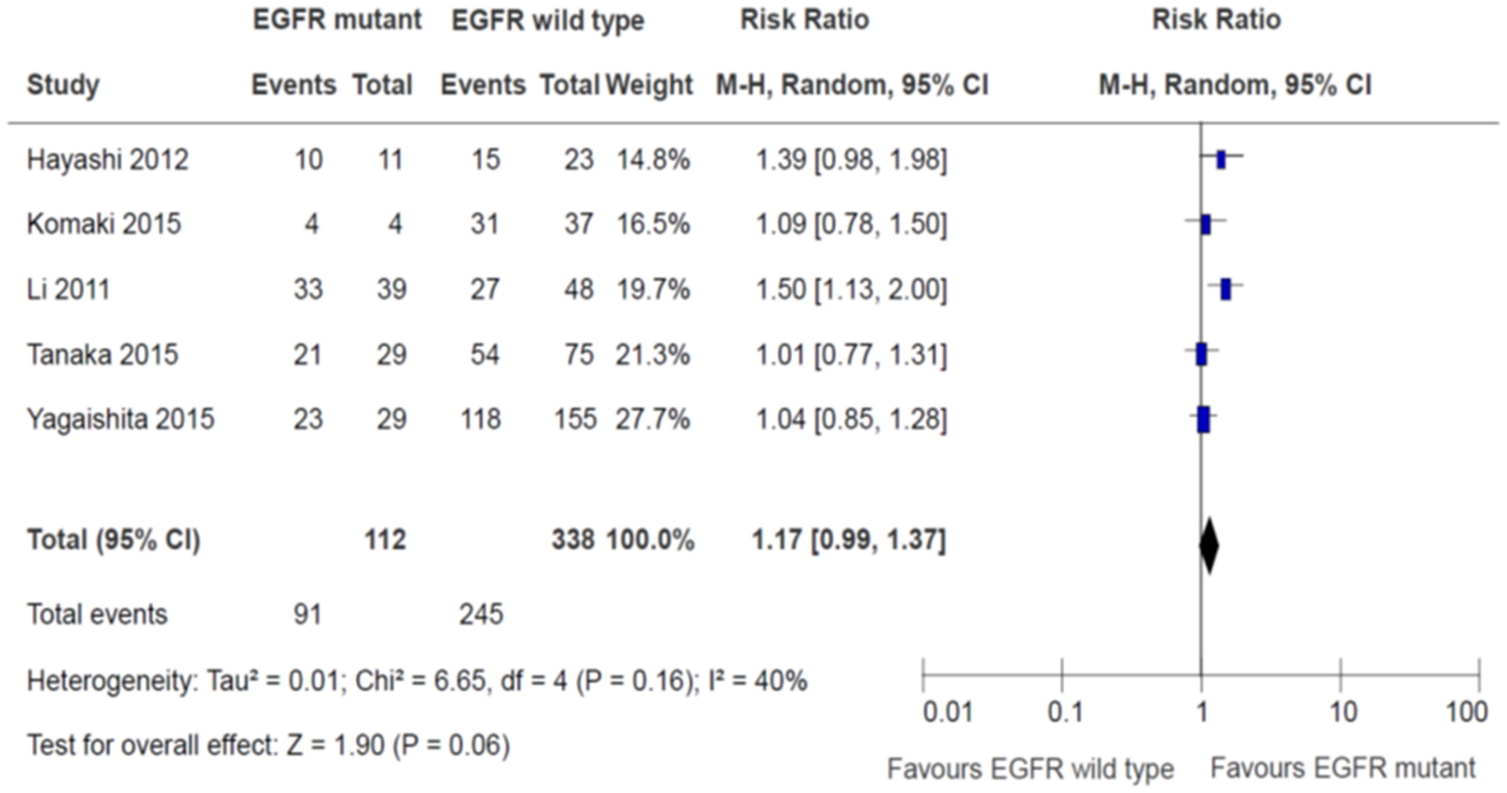
Tumor overall response rates

Five studies reported the rates of the partial and complete tumour response. Four studies defined tumour response as per RECIST 1.1 [11–12, 15–16], while one study used RECIST 1.0 [13]. There was no significant differences in tumour overall response rates (partial + complete response) between EGFR sensitizing mutations and wild type groups (RR 1.17, 95% confidence interval (CI) 0.99 to 1.37, *P* = 0.06; Figure 2). There was no statistically significant heterogeneity in the RR for overall response rate (chi square *P* = 0.16, I^2^ = 40%). There were no significant differences in effects on overall response rates between subgroups defined by study design, use of concurrent chemotherapy or EGFR TK inhibitors (Table 2). The quality of evidence judged by the GRADE approach was deemed to be very low.

**Table 2:** Subgroup effects on overall response rates

Subgroup	Patients	Risk Ratio	95% CI	Interaction *P*
Study Design				
Retrospective analysis of prospective single arm clinical trial	41	1.09	0.78 to 1.50	0.63
Retrospective single institution cohort study	409	1.19	0.98 to 1.45	
Use of concurrent chemotherapy				
Mandatory	145	1.04	0.84 to 1.27	0.22
Not Mandatory	205	1.27	0.99 to 1.63	
Use of EGFR TK inhibitors				
Mandatory	41	1.09	0.78 to 1.50	0.63
Not mandatory	409	1.19	0.98 to 1.45	

### Loco-regional disease recurrence rates

**Figure 3:**
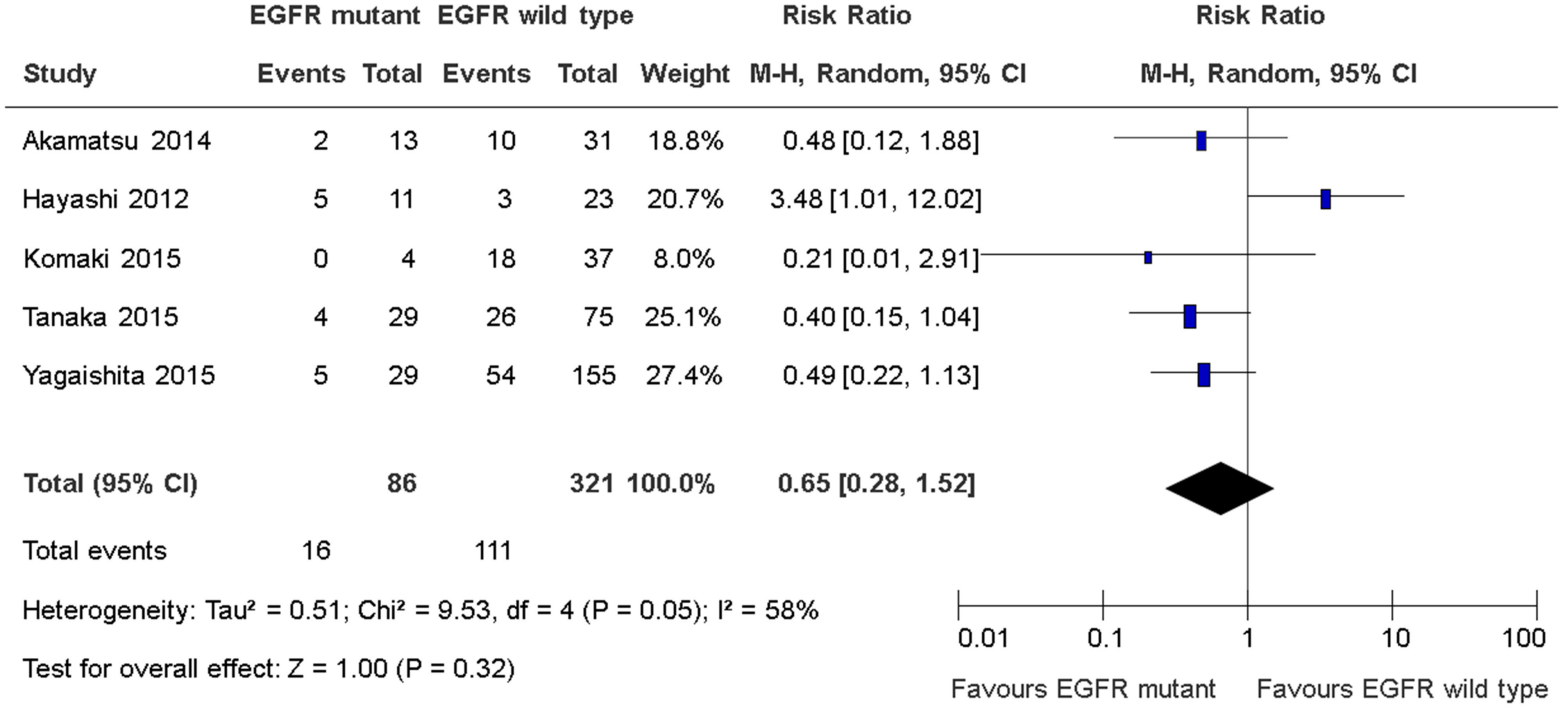
Loco-regional disease recurrence rates

Five studies reported the rates of loco-regional disease recurrence [12–16]. Only one study defined loco-regional disease recurrence as recurrence of disease within the RT fields [16]. The other four studies did not define loco-regional disease recurrence although the results were reported [12–15]. There was no significant differences in loco-regional disease recurrence between EGFR sensitizing mutations and wild type groups (RR 0.65, 95% CI 0.28 to 1.52, *P* = 0.32; Figure 3). There was significant heterogeneity among the trial results (chi square *P* = 0.05, I^2^ = 58%). There were no significant differences in effects on locoregional disease recurrence between subgroups defined by study design, use of concurrent chemotherapy or EGFR TK inhibitors (Table 3). The quality of evidence judged by the GRADE approach was deemed to be very low.

**Table 3:** Subgroup effects on loco-regional disease recurrence rates

Subgroup	Patients	Risk Ratio	95% CI	Interaction *P*
Study Design				
Retrospective analysis of prospective single arm clinical trial	41	0.21	0.01 to 2.91	0.38
Retrospective single institution cohort study	366	0.72	0.29 to 1.80	
Use of concurrent chemotherapy				
Mandatory	145	0.37	0.15 to 0.91	0.25
Not Mandatory	262	0.91	0.26 to 3.10	
Use of EGFR TK inhibitors				
Mandatory	41	0.21	0.01 to 2.91	0.38
Not mandatory	366	0.72	0.29 to 1.80	

### Distant disease recurrence rates

**Figure 4:**
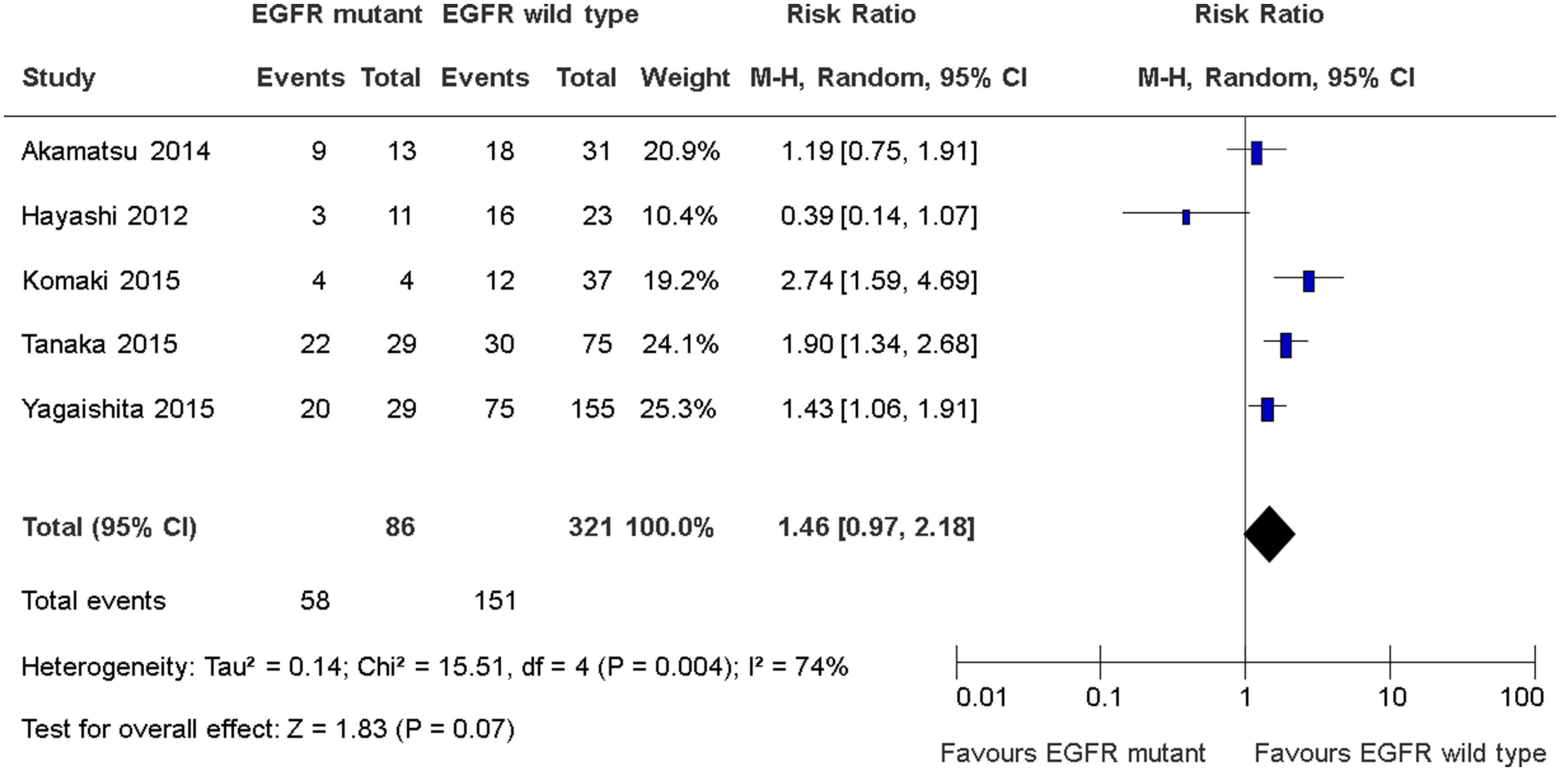
Distant disease recurrence rates

Five studies reported rates of distant disease recurrence [12–16]. Only one study defined distant disease recurrence as recurrence of disease outside the RT fields [16]. The other four studies did not define distant disease recurrence although the results were reported [12–15]. There was no significant differences in distant disease recurrence between EGFR sensitizing mutations and wild type groups (RR 1.46, 95% CI 0.97 to 2.18, *P* = 0.07; Figure 4). There was significant heterogeneity among the trial results (chi square *P* = 0.004, I^2^ = 74%). The effect on disease recurrence rates were greater in study that conduct retrospective analysis of a prospective clinical trial than retrospective single institutional studies (RR 2.74 versus (vs) 1.28, interaction *P* = 0.03); greater in studies that mandate the use of concurrent chemotherapy than studies which did not (RR 2.15 vs 1.05, interaction *P* = 0.03); greater in study that mandates the use of EGFR TK inhibitors than studies which did not (RR 2.74 versus (vs) 1.28, interaction *P* = 0.03) (Table 4). The quality of evidence judged by the GRADE approach was deemed to be very low.

**Table 4:** Subgroup effects on distant disease recurrence rates

Subgroup	Patients	Risk Ratio	95% CI	Interaction *P*
Study Design				
Retrospective analysis of prospective single arm clinical trial	41	2.74	1.59 to 4.69	0.03
Retrospective single institution cohort study	366	1.28	0.85 to 1.92	
Use of concurrent chemotherapy				
Mandatory	145	2.15	1.51 to 3.06	0.03
Not Mandatory	262	1.05	0.61 to 3.06	
Use of EGFR TK inhibitors				
Mandatory	41	2.74	1.59 to 4.69	0.03
Not mandatory	366	1.28	0.85 to 1.92	

### Recurrence free survival

**Figure 5:**
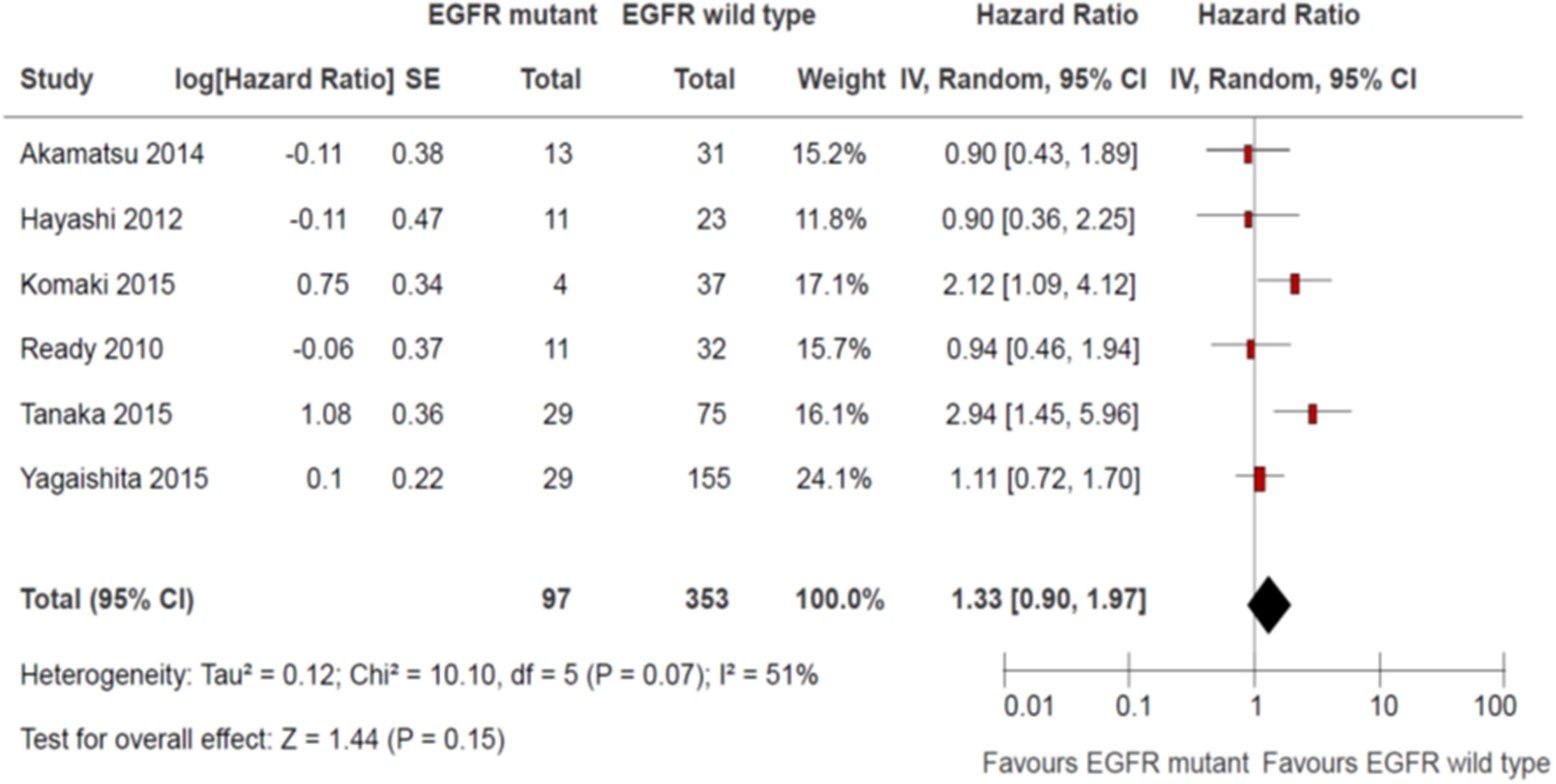
Recurrence-free survival

Six studies reported recurrence free survival [10, 12–16]. Four studies defined recurrence free survival as time from 1^st^ day of treatment (either radiation therapy, chemotherapy or both) to disease recurrence or death [12–13, 15–16]. One study defined recurrence free survival as time from enrolment to disease recurrence or death [10]. One study did not define recurrence free survival although the results were reported [14]. There was no significant differences in disease recurrence free survival between EGFR sensitizing mutations and wild type groups (HR 1.33, 95% CI 0.90 to 1.97; *P* = 0.15; Figure 5). There was significant heterogeneity among the trial results (chi square *P* = 0.07, I^2^ = 51%). The effect on recurrence free survival was greater in studies that mandate the use of concurrent chemotherapy than studies than did not (HR 2.47 vs 1.01, interaction *P* = 0.002). There were no significant differences in effects on recurrence free survival between subgroups defined by study design and use of EGFR TKIs (Table 5). The quality of evidence judged by the GRADE approach was deemed to be very low.

**Table 5:** Subgroup effects on recurrence free survival

Subgroup	Patients	Hazard Ratio	95% CI	Interaction *P*
Study Design				
Retrospective analysis of prospective single arm clinical trial	84	1.43	0.65 to 3.16	0.83
Retrospective single institution cohort study	366	1.29	0.76 to 2.17	
Use of concurrent chemotherapy				
Mandatory	145	2.47	1.52 to 4.02	0.002
Not Mandatory	305	1.01	0.74 to 1.38	
Use of EGFR TK inhibitors				
Mandatory	84	1.43	0.65 to 3.16	0.83
Not mandatory	366	1.29	0.76 to 2.17	

### Overall survival

**Figure 6:**
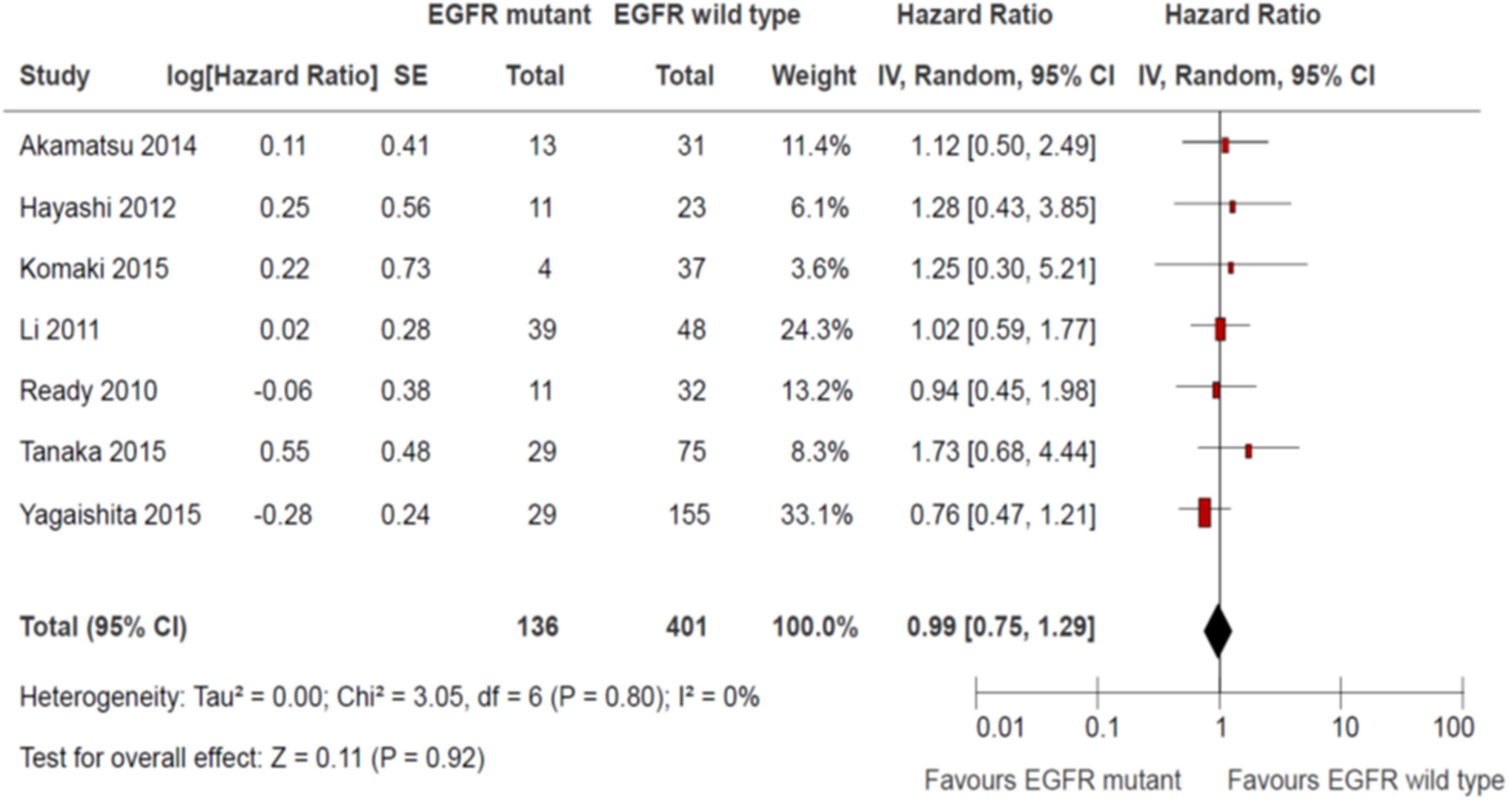
Overall survival

All studies reported overall survival [10–16]. Two studies defined overall survival as time from enrolment to death [10, 14]. Two studies defined overall survival as time from diagnosis to death [11–12]. Three studies defined overall survival as time from 1^st^ day of treatment (either radiation therapy, chemotherapy or both) to death [13, 15–16]. There was no significant difference in overall survival between EGFR sensitizing mutation and wild-type (HR 0.99, 95% CI 0.75 to 1.29; *P* = 0.92; Figure 6). There was no significant heterogeneity among the trial results (chi square *P* = 0.80, I^2^ = 0%). There were no significant differences in effects on overall survival between subgroups defined by study design, use of concurrent chemotherapy or EGFR TKIs (Table 6). The quality of evidence judged by the GRADE approach was deemed to be very low.

**Table 6:** Subgroup effects on overall survival

Subgroup	Patients	Hazard Ratio	95% CI	Interaction *P*
Study Design				
Retrospective analysis of prospective single arm clinical trial	84	1.00	0.52 to 1.94	0.96
Retrospective single institution cohort study	453	0.98	0.73 to 1.32	
Use of concurrent chemotherapy				
Mandatory	145	1.57	0.71 to 3.44	0.22
Not Mandatory	392	0.93	0.69 to 1.24	
Use of EGFR TK inhibitors				
Mandatory	84	1.00	0.52 to 1.94	0.96
Not mandatory	453	0.98	0.73 to 1.32	

### Adverse events

Only one study compared the adverse events outcomes between EGFR sensitizing mutations and wild-type [14]. There was no significant difference in incidence and severity of esophagitis, pneumonitis, skin toxicity and fatigue between the EGFR mutant and wild type groups.

## DISCUSSION

This meta-analysis showed that there was no difference in overall tumour response rate, recurrence free or overall survival between EGFR sensitizing mutations and wild type groups. However, these findings were based on very low quality evidence.

Our results were consistent with published meta-analyses that demonstrated that EGFR sensitizing mutations were not prognostic for localized NSCLC treated with definitive chemoradiotherapy or surgery [17–19]. Ochiai and colleagues performed a systematic review and meta-analysis of three retrospective non-randomized comparative studies to determine the impact of EGFR sensitizing mutations in locally advanced NSCLC treated with concurrent chemo-radiotherapy on patterns of recurrence, recurrence-free and overall survival [17]. They found that there was no difference in disease recurrence, recurrence free and overall survival between EGFR mutated and EGFR wild-type NSCLC, but there was a higher incidence of distant disease recurrence rate and lower incidence of locoregional recurrence rate for EGFR mutated NSCLC. We acknowledged that while the results of our review were mostly consistent with findings of the review reported by Ochiai et al., there are some key differences between the two reviews. Firstly, our population of interest is much broader as we included patients treated with definitive thoracic radiation therapy with or without chemotherapy. Secondly, we included a thorough review on the methodological quality of the included studies as well as an appraisal of the summarized evidence which Ochiai et al. did not perform. Thirdly, we used a random effects model to meta-analysed the results of the included studies, whereas Ochiai et al. adopted a fixed effects model. We believe that it is more appropriate to use a random effects model as there were variation in patient characteristics, utilization rate of concurrent chemotherapy or EGFR TKIs and definition of endpoints among the included studies.

Zhang et al. performed a publication based meta-analysis of 16 studies examining the impact of EGFR sensitizing mutations on disease-free and survival outcomes in resected NSCLC [18]. They found that the presence of EGFR sensitizing mutations was not a prognostic factor in patients with resected NSCLC, but the methodologic quality of the included studies was modest. The findings reported by Zhang et al. were recently confirmed by Shepherd and colleagues who evaluated the prognostic and predictive roles of TP53/KRAS and TP53/EGFR co-mutations in 3,533 patients from the LACE (Lung Adjuvant Cisplatin Evaluation) database of randomized trials of adjuvant chemotherapy versus observation in early stage resected NSCLC [19]. They found that EGFR or KRAS and TP53 tumour suppressor co-mutations had no prognostic effect in resected NSCLC.

Guidelines from the College of American Pathologists, International Association for the Study of Lung Cancer, Association for Molecular Pathology and American Society of Clinical Oncology stated that [20, 21]

1. EGFR mutation testing should be performed at the time of diagnosis for patients with stage IV disease who are suitable for therapy or at a time of recurrence or progression in patients who originally presented with lower-stage disease but were not previously tested.
2. EGFR mutation testing of tumours at diagnosis from patients presenting with stage I to III disease is encouraged but the decision to do so should be made locally by each laboratory in collaboration with its oncology team.

The benefits of upfront EGFR mutation testing in non-metastatic NSCLC include starting treatment early in patients who experience a recurrence as the molecular information is already available. Secondly biopsy taken when patients recurred or progressed may be of borderline quantity and quality and this may affect the results of EGFR testing. Hence testing on initial specimen may be preferable. The downsides of upfront EGFR mutation testing in non-metastatic NSCLC include the additional cost of performing these tests when the results may not be used to guide management in patients who never relapse after curative intent therapies. Secondly, the role of EGFR TKIs in non-metastatic NSCLC has not been established [8]. We are awaiting results of on-going trials such as RTOG 1306 to help determine whether adding upfront EGFR TKIs to standard concurrent chemo-radiotherapy would benefit patients with unresectable locally advanced EGFR mutated NSCLC [22].

We feel that the strengths of this review are as follows:

- It addresses an important clinical question;
- We evaluated the methodologic quality of the included studies as well as the quality of the summarized evidence using published tools
- There was homogeneity among the trial results for overall survival outcomes.

However, the review was limited by:

- Small number of included studies, all of which were not randomized
- Quality of the summarized evidence being very low
- Information gathered from published data rather than individual patient data.

In summary, we conclude that EGFR sensitizing mutations is not a significant prognostic marker for patients with non-metastatic NSCLC undergoing definitive thoracic radiotherapy, with or without chemotherapy.

## MATERIALS AND METHODS

### Study criteria

This meta-analysis incorporated studies comparing the outcomes of interests of patients, with newly diagnosed non-metastatic NSCLC treated with first-line definitive TRT with or without chemotherapy, who were classified according to their EGFR mutation status i.e. EGFR sensitizing mutations versus wild-type status. The EGFR mutations of interest included exon 19 deletions and L858R point mutations in exon 21. We included studies, either in English or Chinese language, where full publication was available.

### Search strategy

Studies were identified by searching MEDLINE from the date of inception onwards to December 2016. The search strategy included the medical subject headings of “radiotherapy”, “lung neoplasms” and “receptor, epidermal growth factor”. The results were then hand searched for eligible trials.

### Selection of studies and data collection

Three reviewers independently assessed the eligibility of abstracts identified by the search. The full text article of any study that appeared to meet the inclusion criteria were retrieved for closer examination. Disagreements were resolved by consensus.

The same three reviewers extracted the data independently using standardized data collection forms. The data retrieved from the reports include publication details, methodological components, study characteristics such as sample size, interventions, duration of follow up and outcome measures. The data extracted from the studies were entered into the Cochrane Collaboration software (RevMan version 4.2.9; http://www.cochrane.org).

### Methodologic quality assessment

Quality assessment of each study was based on the reporting of the study methods and results namely: adequacy in the definition of the study participants with respect to time, place and person, percentage of participants refusing to participate, accuracy in measurement of outcomes, blinding in the measurement of risk factors and outcomes, whether all important risk factors were included in the analysis and percentage of participants not included in the analysis. A global quality score for each study was determined based on the reviewers’ judgement of the importance of these aspects and consequent susceptibility of the results to bias [23].

The quality of a body of evidence for each individual outcome was summarized using the GRADE approach [24]. This approach involved considering the within study risk of bias (methodologic quality), directness of evidence, heterogeneity, precision of effect estimate and risk of publication bias.

### Outcomes measures

The main outcomes of interests were:

1. Tumour overall response defined as complete or partial response as per RECIST criteria [25] or investigator defined criteria
2. Locoregional disease recurrence defined as recurrence of disease proven histologically or radiologically within the radiation fields or investigator defined criteria
3. Distant disease recurrence defined as recurrence of disease proven histologically or radiologically outside the radiation fields or investigator defined criteria
4. Recurrence free survival defined as time from diagnosis or start of any anti-cancer treatment till any disease recurrence proven histologically or radiologically or death from any cause
5. Overall survival defined as time from diagnosis or start of any anti-cancer treatment till death from any cause
6. Adverse events defined as per the Common Terminology Criteria for Adverse Events (CTCAE) version 3.0 or 4.0 [26] or as per investigator defined criteria

### Statistical analysis

For dichotomous outcomes, i.e. tumour overall response, locoregional disease recurrence, distant disease recurrence and adverse events, we extracted the number of patients in each group who experienced the outcome of interest and the number of patients assessed to estimate a risk ratio (RR). The individual study’s risk ratios were pooled using the Mantel-Haenszel method [27]. A RR of more than 1 for tumour overall response suggests an advantage for EGFR sensitizing mutations while risk ratio of more than 1 for the other dichotomous outcomes suggests an advantage for EGFR wild-type.

For time-to-event outcomes, i.e. recurrence free survival and overall survival, the log hazard ratios (HR) and their variances for time-to-event data were estimated using published methods when appropriate summary statistics or Kaplan-Meier curves were reported [28]. The individual study log HR and their variances were then combined using the generic inverse variance method [29]. A HR of less than 1 suggests an advantage for EGFR sensitizing mutations.

Statistical heterogeneity among the included studies’ results was assessed by visual inspection of forest plots, chi-square tests and I^2^ statistic. A *P* value higher than 0.1 for chi-square test and an I^2^ value of lower than 25% was interpreted as signifying a low level of heterogeneity [30]. All meta-analyses were performed with a random effects model.

### Subgroup analysis

Subgroup analyses, determined a priori were performed to determine if the results were influenced by: the use of concurrent chemo-radiotherapy; the use of EGFR TK inhibitors and study design (prospective versus retrospective). Interaction test was used to compare differences between estimates from different subgroups [31].
